# Two-Week Low-Salt Diet Improves Acetylcholine-Induced Microvascular Dilation in Biologically Naïve Psoriasis Patients †

**DOI:** 10.3390/nu17040693

**Published:** 2025-02-14

**Authors:** Ivana Krajina, Mario Štefanić, Ines Drenjančević, Jakov Milić, Nikolina Kolobarić, Vera Plužarić, Stana Tokić, Tatjana Matijević, Maja Tolušić Levak, Maja Kožul, Marija Šola, Ana Stupin, Martina Mihalj

**Affiliations:** 1 Department of Dermatology and Venereology, University Hospital Osijek, J. Huttlera 4, HR-31000 Osijek, Croatia; ivkrajina@mefos.hr (I.K.); vera.pluzaric@kbco.hr (V.P.); matijevic.tatjana@kbco.hr (T.M.); maja.tolusic.levak@mefos.hr (M.T.L.); majakozul@mefos.hr (M.K.); sola.marija@kbco.hr (M.Š.); 2 Department of Physiology and Immunology, Faculty of Medicine, University of Osijek, J. Huttlera 4, HR-31000 Osijek, Croatia; ines.drenjancevic@mefos.hr (I.D.); nbdujmusic@mefos.hr (N.K.); 3 Department of Nuclear Medicine and Oncology, Faculty of Medicine, University of Osijek, J. Huttlera 4, HR-31000 Osijek, Croatia; mario.stefanic@mefos.hr; 4 Catholic Faculty of Theology, University of Zagreb, Vlaska 38, HR-10000 Zagreb, Croatia; jakov.milic@kbf.unizg.hr; 5 Department of Laboratory Medicine and Pharmacy, Faculty of Medicine, University of Osijek, J. Huttlera 4, HR-31000 Osijek, Croatia; stokic@mefos.hr; 6 Department of Infectiology and Dermatovenereology, Faculty of Medicine, University of Osijek, J. Huttlera 4, HR-31000 Osijek, Croatia; 7 Department of Histology and Embryology, Faculty of Medicine, University of Osijek, J. Huttlera 4, HR-31000 Osijek, Croatia

**Keywords:** diet, sodium-restricted, psoriasis, microcirculation, endothelium, laser Doppler flowmetry

## Abstract

**Background/Objectives**: Patients with psoriasis exhibit endothelial dysfunction (EDy), which increases their cardiovascular risk. Excessive salt intake impairs endothelium-dependent vascular reactivity. However, research on EDy in psoriasis has focused primarily on larger vessels, leaving skin microcirculation unexplored. This study aimed to investigate the effects of a low-salt diet (LS diet, ~3.75 g/day) on microvascular reactivity in patients with chronic plaque psoriasis. **Methods**: Laser Doppler flowmetry was used to assess skin microcirculation blood flow in response to vascular occlusion (postocclusive reactive hyperemia, PORH), acetylcholine (acetylcholine-induced dilation, AChID), sodium nitroprusside (SNP-induced dilation, SNPID), and local heating (local thermal hyperemia, LTH). Blood and 24 h urine samples were collected for biochemical and inflammatory parameters and dietary adherence monitoring. **Results**: The results showed significant reductions in systolic (*p* = 0.021) and mean arterial blood pressure (*p* = 0.007) after the LS diet. AChID increased by 16% (*p* = 0.029) regardless of blood pressure changes, especially in normolipemic, normotensive patients without excess visceral fat. Importantly, significant sex differences were observed, with significant improvement in endothelial function observed only in women (*p* = 0.031 vs. *p* = 0.477). PORH did not change significantly; however, a Fourier transformation analysis revealed that the endothelial component of the PORH was significantly improved following the LS diet protocol. The duration of psoriasis negatively correlated with changes in AChID (R = −0.46, *p* = 0.036) and LTH (R = −0.45, *p* = 0.041) after the LS diet, indicating that prolonged low-grade inflammation reduced endothelial function reversibility. Additionally, lower triglyceride, uric acid, and BMI levels were significant predictors of better endothelial function improvement following the LS diet. **Conclusions**: This study supports the beneficial effect of the early introduction of an LS diet in the treatment protocols for patients with psoriasis.

## 1. Introduction

Psoriasis is a chronic inflammatory autoimmune disease, a prototype TNFα–Th17 disease, which represents an individual risk factor for the development of cardiovascular diseases, significantly increasing the morbidity and mortality among affected patients [1,2,3,4]. The concept of cardiovascular morbidity accompanying psoriasis encompasses a spectrum ranging from endothelial dysfunction (EDy) to major adverse cardiovascular events such as myocardial infarction (MI), cerebrovascular insult (CVI), and death [5]. EDy is characterized by impaired vasodilation or enhanced vasoconstriction. The reversibility of endothelial function, along with its role in preceding the onset of serious cardiovascular diseases [6,7], highlights its importance as a target for the prevention of cardiovascular conditions [8].

Over the past four decades, most studies on EDy in psoriasis patients (PPs) have mainly been performed on large-diameter conductive blood vessels [9,10,11,12], typically indicating impaired macrovascular endothelial function in those patients. Additionally, studies have found that macrovascular EDy in PPs is directly related to the disease severity (measured by psoriasis area and severity index, PASI), as well as the level of systemic low-grade inflammation (measured by high-sensitivity C-reactive protein, hsCRP) and the overall cardiovascular (CV) risk of these patients.

However, a study using an animal model of rheumatoid—an autoimmune disease similarly mediated by Th17 cells—has shown that microvascular EDy precedes EDy in large-diameter blood vessels, supporting the hypothesis that microvascular EDy could be an even earlier indicator of cardiovascular risk in such autoimmune inflammatory diseases [13].

Interestingly, there are only very limited data on microvascular EDy in PPs [14], including studies of the cutaneous microcirculation, although its impairment could potentially be important in many diseases, particularly in dermatological diseases, such as psoriasis [12,14]. Alba et al. published the only study in the literature demonstrating attenuated skin microvascular response to localized thermal heating (at 42 °C) measured with laser Doppler flowmetry (LDF) in PPs [15]. Moreover, the LDF method has been well established and shown to be reproducible and sensitive to changes induced by salt intake in the general population, as well as in several high-cardiovascular-risk populations [16]. This makes LDF an ideal tool for assessing blood vessel function and evaluating the impact of a low-salt (LS) diet on endothelial function in a non-invasive and accessible manner.

It has been well established that low-grade inflammation leads to endothelial cell activation [17,18]. A high-salt diet also induces endothelial cell activation and subsequent disbalance of vasodilator and vasoconstrictor metabolites, leading to impaired vasoreactivity [19,20]. An in vitro study showed that TNF-α, in the presence of a high-salt (HS) environment, increases endothelial cell activation in a 3D model of a bifurcating artery under non-uniform shear forces and that this proatherogenic effect of TNF-α was NaCl-concentration-dependent [21], which might be of particular interest in diseases with elevated TNF-α levels, such as psoriasis. A recent finding of NaCl accumulation in the skin/dermis during skin inflammation such as that found in psoriasis and atopic dermatitis, as well as during high-dietary-salt load supports a detrimental effect of NaCl in the skin [22]. Data from a recent study showed that PPs with a disease severity PASI > 5 have increased sodium and water retention in both lesional and non-lesional skin, correlating with disease severity, whereas this effect was not observed in PPs with milder disease forms with PASI < 5 [22].

In addition, evidence provided by molecular and functional studies in PPs linked impaired macrovascular endothelial function to low-grade inflammation and oxidative stress [23,24,25], both of which can be aggravated due to HS intake [17]. Altogether, this finding points out the importance of salt in diseases with elevated TNF-α levels, as is the case of psoriasis. Hence, the hypothesis of the present study was that reduced intake of kitchen salt during a two-week period leads to an improvement in vascular reactivity in the microcirculation of the skin of patients with psoriasis vulgaris.

## 2. Materials and Methods

### 2.1. Patient Population

This study included 28 volunteers with histopathologically proven chronic plaque psoriasis with a PASI ≥ of 5 at the start of this study. The subjects were recruited among patients at the Department of Dermatology and Venereology of University Hospital Osijek (Croatia). The exclusion criteria were age below 18 years, history of other immune-mediated diseases (except autoimmune thyroiditis and psoriatic arthritis), malignancies, current infections, and allergic reactions within 6 weeks prior to study entry. All subjects abstained from local corticosteroid therapy for at least 2 weeks before and during this study. The subjects did not take any systemic drugs with an effect on psoriasis (e.g., methotrexate, retinoids, cyclosporine, systemic corticosteroids, and apremilast) or biological therapy for at least 3 months before and during this study.

**Figure 1:**
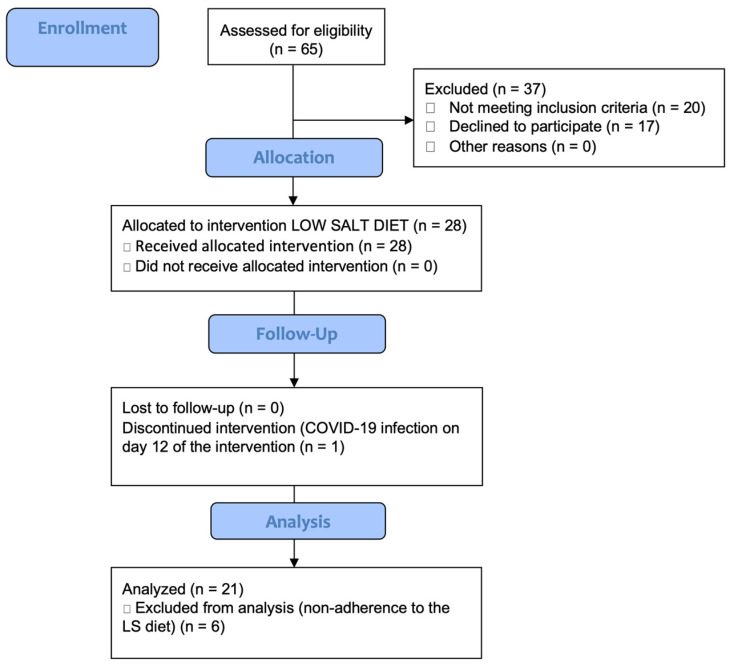
CONSORT diagram. Flowchart of participants throughout this study.

One participant did not complete the study protocol due to COVID infection during the intervention diet, while data from 7 participants were excluded from analysis due to poor adherence to the LS diet, as determined after a 24 h urine sodium analysis. Ultimately, data from 21 participants were analyzed (Figure 1). Written informed consent was collected from each subject. The study protocol and procedures met the standards of the latest revision of the Declaration of Helsinki and were approved by the Ethics Committee of the Faculty of Medicine Osijek (class: 602-04/21-08/07, number: 2158-61-07-21-153 and class: 602-04/23-08/03, number: 2158-61-46-23-41), as well as the Ethics Committee of the University Hospital Osijek (number: R2-13170/2020). This research was conducted at the Department of Dermatology and Venereology of the Osijek University Hospital and at the Department of Physiology and Immunology of the Osijek Faculty of Medicine University of Osijek. This research was part of a clinical trial that investigated the effect of an LS diet on Th17-mediated inflammation and vascular reactivity and is registered at ClinicalTrials.gov (ID NCT05892640).

### 2.2. Study Protocol

This was a prospective self-controlled clinical cohort study with a single group of subjects with chronic plaque psoriasis, where all subjects (N = 28) were instructed to follow a 2-week low-salt (LS) diet, with a daily salt intake of 3.75 g (e.g., 1500 mg sodium) according to the Dietary Approaches to Stop Hypertension eating plan (DASH; US department of Health and Human Services; 2006). The study protocol consisted of two visits, at baseline and on the last day of the 14-day LS intervention. The LS diet protocol was explained to the subjects in person and in writing at the baseline visit. The severity of psoriasis was determined using the Psoriasis Area and Severity Index (PASI) [26].

### 2.3. Analysis of the 24 H Urine Sample

In order to prove adherence to the given dietary protocol, all subjects had their 24 h urine samples collected prior to both study visits. The samples were analyzed for sodium, potassium, urea, and albumin concentrations and creatinine coefficient at the Department of Clinical Laboratory Diagnostics, University Hospital Osijek. The following formula was used to calculate the daily salt intake: 1 g of salt (NaCl) = 393.4 mg Na = 17.1 mmol Na [18].

### 2.4. Measurement of Hematological and Biochemical Parameters from Whole Blood and Serum

The blood analyses were performed according to standardized, IVD-certified routine protocols as follows: complete blood count with differential (CBCD) from the whole blood sample collected during both study visits and determined using the Sysmex XN 2000 (Sysmex Corporation, Kobe, Japan), and biochemical parameters (glucose, urea, creatinine, uric acid, high-sensitivity C-reactive protein (hsCRP), cholesterol, triglycerides, high-density lipoprotein (HDL), low-density lipoprotein (LDL), calcium (Ca), sodium (Na), and potassium (K)) were measured using the DxC700 AU biochemical analyzer (Beckamn Coulter, Brea, CA, USA). Folic acid and vitamin B12 were determined in serum samples with the cobas e601 analyzer (Roche, Basel, Switzerland) and fibrinogen activity in plasma with the BCS XP (Siemens Healthcare, Marburg, Germany) at the Institute for Clinical and Laboratory Diagnostics of the University Hospital Osijek.

### 2.5. Evaluation of Skin Microcirculatory Blood Flow and Microvascular Reactivity

Laser Doppler flowmetry (LDF) (MoorVMS-LDF, Axminster, UK) was used to measure blood flow and blood vessel reactivity in the skin microcirculation in response to four stimuli in the Laboratory for Clinical Physiology and Physiology of Sport, Dept. of Physiology and Immunology, Faculty of Medicine Osijek, Osijek, Croatia. Blood flow and microvascular reactivity were measured on healthy, non-lesional skin of the volar portion of both forearms, 13–15 cm from the wrist, during both study visits as described previously [18,27]. In short, the overall change in microvascular reactivity was determined by measuring the changes in blood flow in response to a 1 min vascular occlusion that induced postocclusive reactive hyperemia (PORH). The spectral analysis of the PORH-LDF signal was performed with the original software MoorVMS-PC v4.0. This software measures the power spectral density of the LDF-PORH signal, and the final result was the percentage of the sum of the power signal values in the frequency bands related to endothelial activity (0.008–0.02 Hz), sympathetic activity (0.02–0.05 Hz), myogenic activity (0.05–0.15 Hz), respiratory activity (0.15–0.6 Hz), and cardiac activity (0.6–2.0 Hz). In addition, iontophoresis of acetylcholine (ACh) and local heating of the skin to 42 °C were used to investigate endothelium-dependent mechanisms of vascular reactivity. Acetylcholine-induced dilation (AChID) represents endothelium-dependent vasodilation in general, while local heating (LTH) specifically represents NO-mediated endothelium-dependent mechanisms of vasodilation, as NO is responsible for 60–70% of the LTH plateau response. Iontophoresis of sodium nitroprusside (SNP), which causes SNP-induced dilation (SNPID), was used to assess endothelium-independent vasodilation. The skin iontophoresis controller (moorVMS-IONTO, Moor instruments, Axminster, UK) was used for iontophoresis protocols, and the skin heater controller (moorVMS-HEAT, Moor instruments, Axminster, UK) was used for the heating protocol.

### 2.6. Blood Pressure Measurements and Anthropometric Measurements

At both study visits, systolic (SBP) and diastolic (DBP) blood pressure was measured with an automatic oscillometric sphygmomanometer (OMRON M3, OMRON Healthcare Inc., Osaka, Japan). The median of three consecutive measurements was calculated. Mean arterial pressure (MAP) was derived using the formula: MAP = ((2 × DBP) + SBP)/3 (mmHg). Body height, weight, waist, and hip circumferences were measured, and body mass index (BMI) and waist-to-hip ratio (WHR) were calculated accordingly [28].

### 2.7. Statistical Analysis

The normality of the distribution of the observed numerical variables was tested using the Shapiro–Wilk test. Continuous data are presented as arithmetic mean ± standard deviation (SD) for variables with a normal distribution or as median and interquartile range (IQR) for variables that did not follow a normal distribution. The categorical data were summarized by absolute and relative frequencies. Fisher exact test was used to analyze contingency tables. Given the total sample size and non-Gaussian paired differences, the non-parametric Wilcoxon rank sum test was adopted for pairwise comparisons. The shift between the two distributions (treatment effect) was quantified using the Hodges–Lehmann estimator [29,30]. For independent, between-group comparisons, the Mann–Whitney test was used. The association between continuous variables was assessed using Spearman’s correlation test. A two-tailed *p* < 0.05 was considered significant. Non-parametric (Spearman’s) partial correlations were tested by analyzing probability-scale residuals [31,32]. For multiple predictors, the predictive value (relative importance) of each covariate was jointly assessed using the SHAP (Shapley additive explanations) value decomposition, a well-established method of model-agnostic, interpretable machine learning. To this end, the methodology of Aas, Jullum, and Løland [33] was used by applying Monte Carlo-based conditional inference trees (4 batches, 2500 samples/batch) [34]. For numeric-only features, model estimation and visualization were performed using tree-based gradient boosting (*xgboost*, empirical conditional distribution, 5000 rounds) and stacked force plots (hierarchical clustering, Ward.D method), respectively [35].

All statistical analyses were performed in R 4.3.2 (www.r-project.org) and Python 3.9.0 (https://www.python.org) using the following packages: asht (v1.0.1), corrplot (v0.9.2), ComplexHeatmap (v2.16.0), cowplot (v1.1.1), DescTools (v0.99.40), ggraph (v2.1.0), ggpubr (v0.6.0), ggstatsplot (v0.12.3.9000), Hmisc (v5.1-1), igraph (v1.5.1), magrittr (v2.0.3), pandas (v2.2.2), PResiduals (v1.0-1), RColorBrewer (v1.1-3), reshape2 (v1.4.4), scales (v1.3.0), scikit-learn (v1.5.1), SHAP (v0.38.1), SHAPforxgboost (v0.1.3), shapr (v0.2.3.9200), shiny (v1.7.5.1), stats (v4.3.1), tidyverse (v2.0.0), and xgboost (v1.7.7.1). The codes needed to replicate the results will be shared upon reasonable request to the corresponding author.

Power calculations were performed using the G*Power Calculator program v3.1.9.4 [36]. Assuming a two-tailed type I error rate of α = 0.05 and an unknown parent distribution (minARE option), the sample size required to achieve a minimum of 80% power to detect a medium-to-large effect size (Cohen’s dz ≥ 0.7, standardized mean difference) was 21 or more (Wilcoxon signed-rank test).

## 3. Results

### 3.1. Descriptive Analysis

All subjects (n = 21) were adults with histopathologically proven chronic plaque psoriasis who followed a 2-week LS diet. The data from the questionnaire on the subjects’ characteristics, habits, and health are shown in Supplementary Table S1. Twelve patients (60%) met the NCEP ATP III guidelines on central obesity, eleven had a record of hyperlipidemia, whereas four patients had metabolic syndrome based on both the ATP III and IDF2005 criteria. There was no significant overlap between central obesity and hyperlipidemia (Fisher’s exact *p* = 0.2), but abdominally obese patients had higher mean arterial pressure on regular-salt diet (RS) [102 (98–105) vs. 93 (85–97) mmHg, *p* = 0.0094, Mann–Whitney test]. Seven patients (one-third) had a clinical history of hypertension requiring antihypertensive therapy. Hypertensive participants were significantly older [median (IQR), 63 (50–73) vs. 37 (31–52) years, *p* = 0.015] and had higher serum triglycerides [1.81 (1.43–2.22) vs. 1.17 (0.63–1.43), mmol/L, *p* = 0.01], lower creatinine clearance [136 (114–139) vs. 165 (150–177) mL/min, *p* = 0.0045], and similar MAP [102 (98–103) vs. 95 (89–101) mmHg, *p* = 0.093, Mann–Whitney test] compared to their normotensive counterparts.

### 3.2. Anthropometric, Haemodynamic, and Biochemical Changes in Response to LS Diet

**Figure 2:**
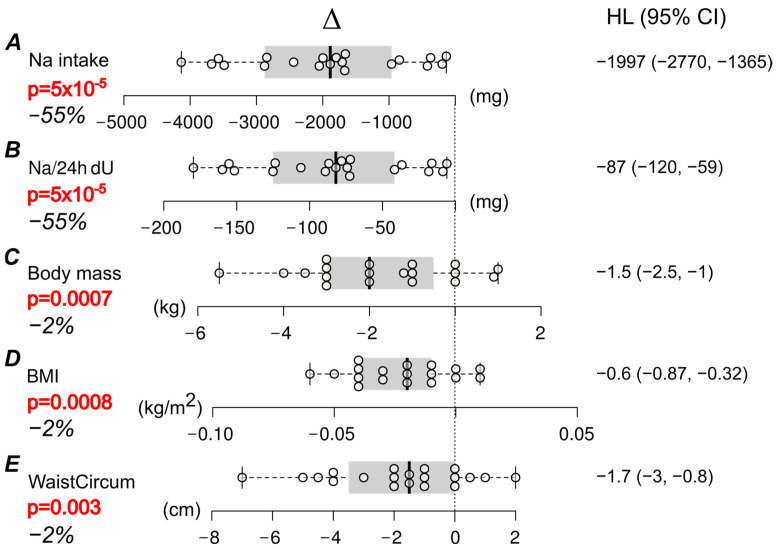
Pairwise comparisons, calculated sodium (Na) intake (**A**), sodium (Na) excretion in 24 h urine (Na/24 h dU, panel (**B**)), body mass (**C**), body mass index (BMI, panel (**D**)), and waist circumference (WaistCircum, panel (**E**)), before and after a low-salt (LS) diet. Δ represents the pairwise difference (LS–regular diet), with unbiased Hodges–Lehmann (HL) estimates of treatment effects and their respective 95% confidence intervals (CI). Box plots show the median (solid vertical line), interquartile range (shaded area, IQR), and whiskers extending to 1.5 × IQR (upper-lower quartile). All plots are aligned to zero (no response, dotted line), and each dot corresponds to one participant. Significant differences are indicated by bold red *p*-values (Wilcoxon paired test). % denotes a percent change from baseline (median).

Adherence to the LS diet was confirmed by a reduction in 24 h natriuresis in all subjects (Figure 2, Table 1). Sodium intake on the RS was significantly higher in men (12.36 ± 3.46 g of salt/day (e.g., 4942 ± 1385 mg of sodium/day)) than in women (8.68 ± 2.37 g of salt/day (3471 ± 948 mg of sodium/day); Wilcoxon signed-rank test, *p* = 0.014).

BMI, body weight, and waist circumference decreased significantly after the 2-week LS diet (Figure 2, Table 2). The LS diet did not lead to a significant change in hip circumference or WHR.

**Figure 3:**
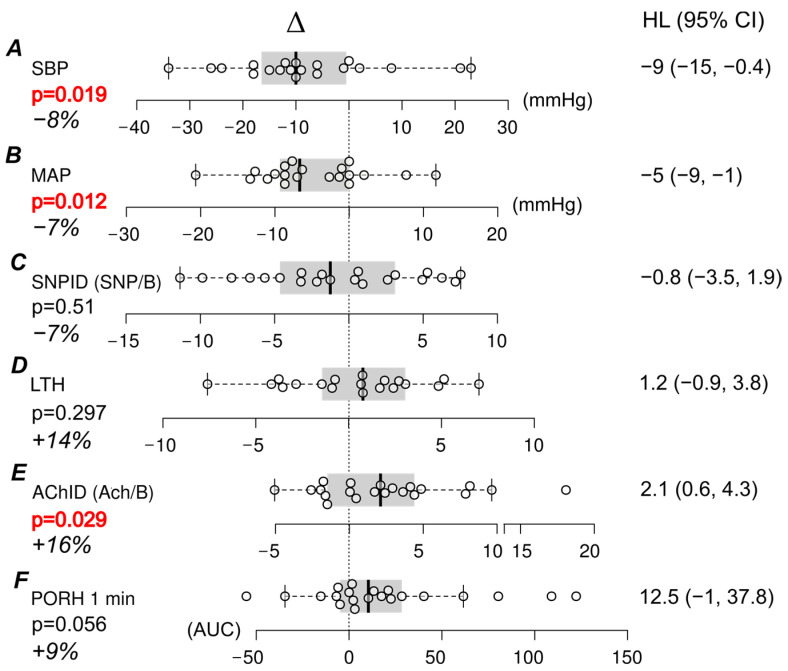
Pairwise comparisons, changes in systolic blood pressure (**A**), mean arterial pressure (**B**), and Laser Doppler flow measurements (**C**–**F**) after a 14-day low-salt (LS) diet. Δ denotes pairwise differences (Δ, LS–regular diet). HL represents unbiased, Hodges–Lehmann estimates of treatment effects, and their respective 95% confidence intervals (CI). Box-and-whisker plots denote the median (solid vertical line) and interquartile range (shaded area, IQR). Whiskers extend to 1.5 × IQR (Q3–Q1). All plots are aligned to zero (no response, dotted line). Each dot corresponds to one participant. Significant differences are indicated by bold red *p*-values (Wilcoxon paired test). SBP—systolic blood pressure; MAP—mean arterial pressure; SNPID (SNP/B)—dilation induced by sodium nitroprusside (flow after iontophoresis with sodium nitroprusside/basal flow); LTH—local thermal hyperemia; heat flow (increase)—increase in flow after local heating of the skin to 42 °C; AChID (Ach/B)—acetylcholine-induced dilation (flow after acetylcholine iontophoresis/basal flow); PORH 1 min AU—post-occlusive reactive hyperemia after 1 min of occlusion (area under the curve). % denotes a percent change from baseline (median).

Following the LS diet, there was a significant decrease in SBP and MAP values (Table 2, Figure 3A,B). The estimated treatment effect size was similar in both hypertensive (treated) and normotensive individuals. (−10.1 vs. −9.5 mmHg, systolic BP, median Hodges–Lehmann estimates, *p*-value for equivalence = 0.66, Wilcoxon test).

After the LS diet, there was no significant change in the counts of blood cells in all three lines (Table 1). The subjects had normal renal function and normal serum electrolyte levels. The LS diet led to an increase in serum creatinine, although the values were within the reference intervals for both diets. The serum urea, sodium, and potassium concentrations were similar. The LS diet marginally increased the serum calcium concentrations, although both values were within the reference range (Table 1). Median disease activity significantly decreased after the 14-day LS diet, as judged by PASI [−1.2 (−1.9, −0.4), Hodges–Lehmann estimate (95% CI), *p* = 0.0011, Table 1].

### 3.3. Microvascular Reactivity in Relation to an LS Diet

No significant change in SNPID or LTH was observed (Table 2, Figure 3C,D).

By contrast, we found significantly improved AChID of the forearm microcirculation after the LS diet, which is considered an endothelium-dependent process (Figure 3, Table 2). AChID increased independently of blood pressure since no correlation was found between the change in AChID and the change in SBP (Spearman’s *R* = 0.11, *p* = 0.63), DBP (*R* = 0.31, *p* = 0.18) and MAP (*R* = −0.28, *p* = 0.24). Overall, a 16% increase from baseline was seen over 14 days (Figure 3), suggesting a modest modulation by short-term salt restriction. Normolipemic patients and patients without excess visceral fat contributed most to this effect, whereas no response to the LS diet was seen when studying their counterparts (Supplementary Table S2, Supplementary Figure S1).

**Figure 4:**
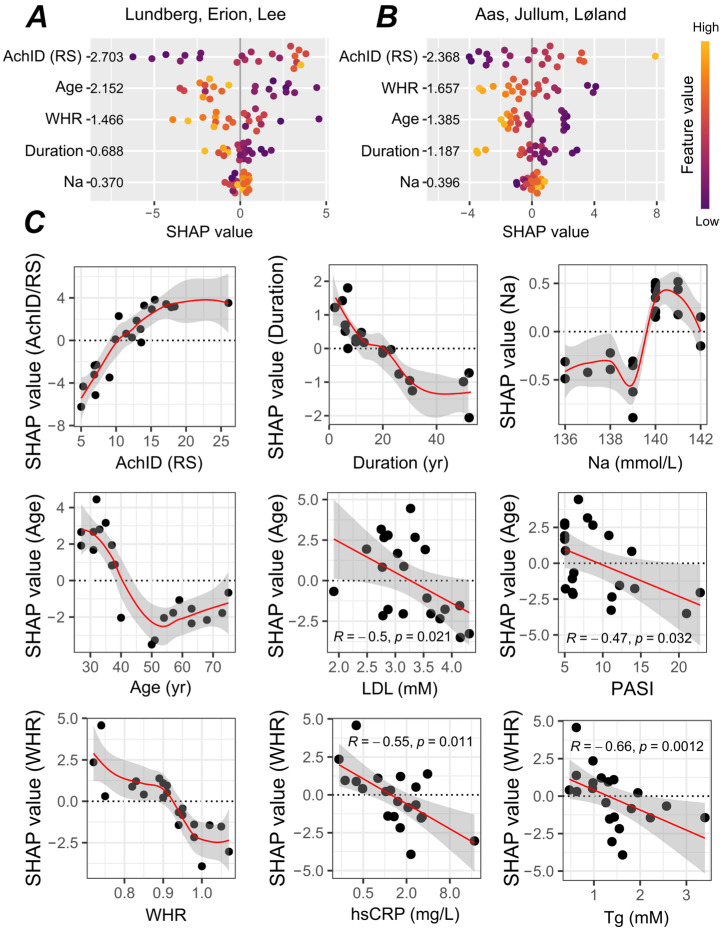
Acetylcholine-induced dilation measured after a 14-day low-salt diet, the predictive value (relative contribution) of each covariate (Shapley value decomposition). (**A**,**B**) Summary of SHAP values for every predictor considered in this analysis; headers (abbreviated authors’ names) denote two different SHAP prescriptions [boosted trees, Refs [35], panel (**A**), and [33], panel (**B**)]. Features were ranked vertically by their mean absolute SHAP values over all observations (bold on the right of the variable names; the larger the absolute SHAP value, the greater the importance of the predictor for model’s output). Clusters of dots around the SHAP value of zero indicate a small impact on model output; each dot corresponds to one participant. The color is scaled to the feature value from low to high. (**C**) Partial dependence plots for panel A, showing how the impact of feature changes as the feature value changes. The feature of interest is represented along the horizontal axis, while the corresponding SHAP values are plotted on the vertical axis. For the average first-order effect—the direct contribution of a single feature independent of the other features—a non-parametric locally weighted running line smoother (LOESS) was fitted to visualize trends in the data (top row-left column, shade represents standard error). For the second-order effects, which illustrate how the impact of a certain feature varies depending on the values of other features, a linear fit (95% confidence interval) is shown since LOESS did not provide any additional information. AChID—acetylcholine-induced dilation; RS –regular-salt diet; Na—sodium; WHR—waist-to-hip ratio; PASI—psoriasis area and severity index; hsCRP—high-sensitivity C-reactive protein; LDL—low-density lipoprotein; Tg—serum triglycerides; mM—mmol/L.

On closer look, other factors were also associated with this modulation. Aged skin showed attenuated microvascular response to ACh on both diets (Supplementary Figure S2A,B). As a result, basal and post-treatment AChID were highly interrelated (Supplementary Figure S2C), indicating a strong memory of initial conditions up to the 14th day of the LS diet. In addition, a long-standing disease and an (expectedly) higher WHR was associated with blunted AChID on the LS diet (Supplementary Figure S2D,E). On the contrary, serum sodium levels weakly tracked AChID: the higher the basal sodium levels, the higher the AChID on the LS diet (Supplementary Figure S2F). Figure 4 summarizes the SHAP values for every potential covariate derived from this correlation analysis, illustrating their respective predictive impact in multivariate settings. Initial microvascular reactivity to ACh, age, and WHR appeared to be among the more important predictors of cutaneous AChID at 2 weeks of the LS diet, followed by disease duration (Figure 4A). By contrast, serum sodium levels were tightly regulated, translating into very little overall contribution to the model’s predictions.

In the time domain, the overall effect of salt restriction on PORH 1 min (paired differences, 14-day LS diet vs. RS diet) was not significant (Figure 3, Table 2). The result, however, varied by hypertension status, central obesity, and hyperlipidemia (but not other patients’ characteristics). Patients receiving long-term antihypertensive treatment (a vasodilating β-blocker—bisoprolol or nebivolol—with or without the addition of the ACEI/ARB) had significantly higher basal PORH 1 min compared to those who did not use antihypertensive drugs (Supplementary Table S2, *p* = 0.039, Mann–Whitney test). No further change in PORH 1 min was observed on an LS diet in this group (Supplementary Figure S1A). By contrast, a significant increase in PORH 1 min was observed after an LS diet in patients not taking antihypertensive drugs (Supplementary Table S2, Supplementary Figure S1A). Since all the patients who had been diagnosed with hypertension were medicated, we duly note the inseparability of their respective effects in our cohort.

A robust increase in PORH 1 min. in response to the LS diet was also found in patients free from central obesity and hyperlipidemia but was absent in those with abdominal obesity or hyperlipidemia (Supplementary Figure S1B, Supplementary Table S2). Thus, despite the large dispersion of ΔPORH values, a significantly increased cutaneous PORH in response to short-term salt restriction seems to stand out, particularly in normotensive, non-obese, and normolipemic patients.

In the frequency domain, as expected, β–blocking agents significantly increased the relative contribution of the sympathetic component to the LDF spectrum on RS diet [14.6 (12.3–16.8) vs. 10.2 (7.3–12)%, raw signal, treated vs. untreated subjects, *p* = 0.017, Mann–Whitney test]. The spectral analysis of the LDF-PORH signal (pooled testing, irrespectively of the antihypertensive drugs) showed that the LS diet in PPs significantly increased the proportion of the power signal (% of total signal) in the frequency subinterval related to endothelial activity [Supplementary Table S3, Hodges–Lehmann (95% CI) = 5.2 (0.2, 12.4)%], but not in those related to myogenic, cardiac, respiratory, or local vascular sympathetic activity. Restricting the analysis to normotensive individuals further strengthened this result (Supplementary Table S2). Again, an apparent variation in dietary effect by central obesity and dyslipidemia was noticed. In particular, an increase in endothelial signal was more readily observed in non-obese compared to centrally obese subjects after an LS diet. The same was true for patients without a record of hyperlipidemia (Supplementary Table S2, Supplementary Figure S1).

To further analyze these traits, we next draw dependence plots to explore how the feature contribution changes with the feature value (e.g., main effects). In addition, we plotted the SHAP values against different covariates, looking for non-trivial, residual associations (second-order effects).

As shown in Figure 4C, a non-linear relationship between the WHR, advancing age, and their corresponding SHAP values was found. Below the value of 0.9 (which coincidently meets the WHO’s definition of abdominal obesity), the contribution from WHR was mostly limited and stable, but higher values of WHR had an increasingly negative impact on the model’s prediction of AChID on the LS diet. In this region, the exact trend was related to serum triglycerides and CRP levels: the higher the triglycerides and CRP, the greater the negative impact of WHR. For age, a detrimental effect emerged in the fifth decade, particularly in patients with the highest serum LDL–cholesterol and initial disease activity, whereafter its negative contribution tends to stabilize. For initial AChID and disease duration, no deviation from proportionality was observed, but for decades-long diseases (>20 yrs), the impact of duration could no longer be separated from age, resulting in a flat evolution of SHAP values. A detailed account of the individual SHAP predictions is available in Supplementary Figure S3. Notably, the final model was largely insensitive to the addition of extra variables: there was little or no evidence of an independent effect for changes in BMI and MAP (sensitivity analysis, Supplementary Figure S4).

**Figure 5:**
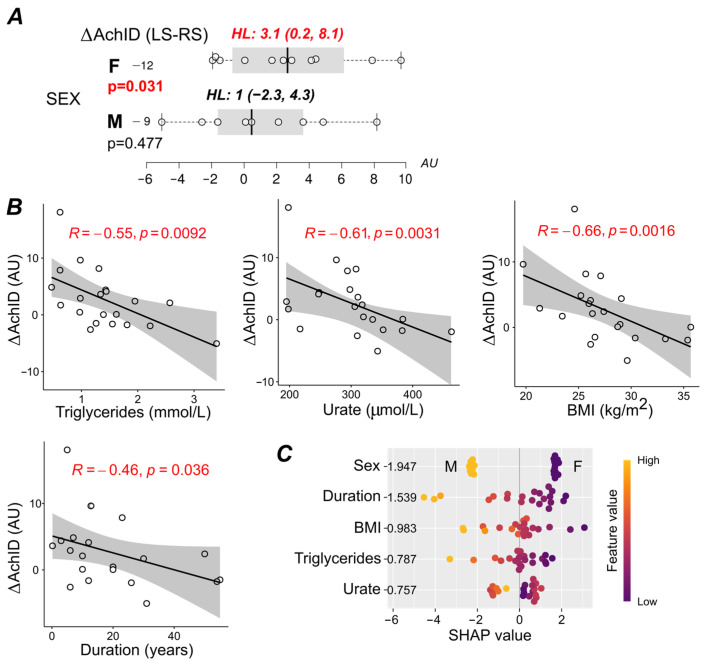
Predictors of change in acetylcholine-induced dilation (AChID) between baseline values on a regular diet and values after a 14-day low-salt diet. (**A**) The interaction of sex and diet (F/M female/male). Δ represents pairwise differences (low-salt–regular diet). HL denotes Hodges–Lehmann estimates of treatment effects; 95% confidence intervals are given in parentheses. *p*-values belong to the Wilcoxon paired test. Box plots with median (solid vertical line), interquartile range (shaded area, IQR), and whiskers extending to 1.5 × IQR (upper-lower quartile). Each dot represents one participant. Significant results (*p* < 0.05) are indicated by bold red *p*-values. (**B**) Correlation analysis. Scatter plot showing variations in treatment effect across the selected metabolic and clinical features. R denotes Spearman’s correlation coefficient. The black line represents a linear model fit (least squares method), whereas the shaded region corresponds to a 95% confidence interval. (**C**) Predictive importance of each covariate and summary of SHAP values for every predictor considered in this analysis. Features were ranked in decreasing order by their mean absolute SHAP values over all observations (bold on the right of the variable names; the larger the absolute SHAP value, the greater the importance of this predictor for the model’s output). Each dot corresponds to one participant. The color is scaled to the feature value from low to high. AChID—acetylcholine-induced dilation (flow after acetylcholine iontophoresis/basal flow); BMI—body mass index.

To further extend those findings, partial Spearman correlations for the net change in AChID on the LS diet after controlling for the baseline have been tested (Supplementary Figure S5). For ΔAChID, significant sex differences were observed. In women, the change in AChID after the LS diet was statistically significant compared to the RS diet, while no such change in AChID occurred in men (Figure 5A). The apparent dimorphism was not related to different salt intake by sex. Other potential predictors for the positive change (increase) in AChID after the LS diet included a recent-onset disease and a highly entangled combination of lower serum triglycerides, lower serum urate, and lower BMI values at baseline (Supplementary Figure S6, Figure 5B). In multivariate settings, sex and disease duration were top-ranked features, but we found the impact of BMI, Tg, and serum urate too tightly correlated to make a solid inference (Figure 5C).

For ΔPORH-ET, hypertension (Supplementary Table S2, hypertensive vs. normotensive patients, *p* for equivalence of two median treatment effects = 0.032), Tg (*R* = −0.67, *p* = 0.00083), WHR (*R* = −0.58, *p* = 0.006), and BMI (*R* = −0.49, *p* = 0.023) were all inversely related to the treatment effect. Of these, hypertension emerged as the top predictor by a large margin (Supplementary Figure S7A); by contrast, the BMI appeared mostly redundant in the multivariate assessment, particularly in patients with a BMI < 25 kg/m^2^. For serum Tg, a negative impact emerged beyond the 1.5 mmol/L threshold; for WHR, the results matched those for AChID under salt restriction, providing an independent replication of trends and limits (WHR > 0.9, Supplementary Figure S7B).

## 4. Discussion

This study examined the effects of a reduction in dietary salt intake during a period of 2 weeks on skin microvascular reactivity in patients with psoriasis. The most important finding of the present study is that a short-term LS diet is associated with significantly improved endothelium-dependent vasodilation (in response to acetylcholine) independently from changes in blood pressure. In addition, this effect was more prominent in women and participants with shorter disease courses, with a decrease in daily salt intake.

In most parts of the world, salt intake clearly exceeds the WHO recommended intake of less than 5 g of salt per day (2000 mg of sodium per day). There is a lack of epidemiological studies on salt consumption in PPs. The subjects suffering from psoriasis in this study also had a higher salt intake than recommended, with 10.26 ± 3.37 mg salt per day (4102 ± 1348 mg of sodium per day) in their RS diet, whereby men consumed more salt than women. It is generally recognized that HS intake is an important risk factor for the development of high blood pressure [37,38]. To our best knowledge, this is the first study to investigate the influence of an LS diet on the hemodynamic parameters in PPs. In this study, SBP, DBP, and MAP decreased significantly following an LS diet in normotensive PPs, confirming the previously known data obtained in the general population.

EDy is present in PPs [12,39,40]. Here, our study demonstrated that an LS diet intervention improved endothelium-dependent microvascular reactivity in PPs. As already mentioned, most studies on vascular reactivity in PPs have been carried out on the conducting vessels [10,41,42]. However, as indicated by a study on an animal model of rheumatoid arthritis, an autoimmune disease immunopathologicaly related to psoriasis, microvascular EDy seems to precede EDy in larger vessels, suggesting that microvascular EDy may be an even earlier indicator of cardiovascular risk in such inflammatory diseases [13]. However, only one study assessed microvascular reactivity in psoriasis, demonstrating endothelium-dependent EDy in PPs. Thus, our knowledge on microcirculation in psoriasis is scarce [15].

In this study, there was a significant recovery of endothelium-dependent vascular reactivity in the form of a 16% increase in dilation in response to acetylcholine (AChID). Interestingly, AChID values at the second visit, as well as the delta change in AChID values after the LS diet correlated negatively with psoriasis duration. Since longer disease duration suggests a longer cumulative duration of low-grade inflammation in the body, it would be valuable to further investigate this concept on the reversibility of endothelial function by reducing salt intake. A study on hypertensive postmenopausal women showed that women whose endothelial function was improved after the administration of antihypertensive drugs had less cardiovascular events [43]. These findings suggest that reversibility of endothelial function might be linked to a favorable cardiovascular prognosis. In the context of our study results, this would mean that an early introduction of LS diet in PPs soon after diagnosis might improve cardiovascular prognosis in our patients, although a study with a longer follow-up would be necessary to make such conclusions. The increase in PORH values was not statistically significant, although at the very edge (*p* = 0.056), except in normotensive, non-obese, and normolipemic patients. A possible explanation for this might be that hypertension, obesity, and hyperlipidemia are per se associated to endothelial dysfunction [44,45], which might be a reason why PORH in these PPs are less responsive to changes in salt intake. However, there was significant increase in the proportion of the PORH power signal (% of total signal) in the frequency subinterval related to endothelial activity (*p* = 0.036), which indicates that the observed increase in PORH following an LS diet could be attributed to the increase in endothelial activity in psoriasis patients. LTH was used to specifically evaluate NO-mediated endothelium-dependent vasodilation. The 14% increase in LTH between the two visits was not significant. However, due to the negative correlation of psoriasis duration with the change in LTH between the two study visits and the LTH values after the LS diet, it seems that PPs who suffer longer from psoriasis have decreased NO bioavailability after the LS diet, which could be due to the long-term changes in the blood vessels caused by psoriasis. Since there was an increase in AChID and no statistically significant increase in flow with LTH, it can be concluded that the restoration of vascular function in the skin microcirculation of PPs after an LS diet is mainly mediated by endothelium-dependent factors other than NO, such as neurotransmitters, endothelium-derived hyperpolarizing factors (EDHFs), and others. However, a limitation of the present study is that none of the potential mediators were measured. Interestingly, EDHF does not have a major impact on microvascular function under normal circumstances [46], but in states of reduced NO bioavailability, it becomes an important mechanism [47]. The fact that there was no change in SNPID values suggests that vascular smooth muscle cells were not affected by salt intake.

The effect of a short-term diet with increased or decreased salt intake on vascular reactivity is already a proven concept. The effect of a short-term HS diet on the deterioration of endothelium-dependent EDy has been clearly demonstrated by our and other research groups in healthy volunteers, competitive athletes, and cardiovascular patients [17,48]. On the other hand, reduced salt intake led to an improvement in endothelium-dependent vasodilation in normotensive study participants independent of blood pressure value changes [49].

Some studies have found a correlation between the degree of EDy and the clinical severity of psoriasis as measured by the PASI score [50,51] or disease duration [9], but there are also conflicting reports [52]. In our study, no significant correlation was found between the degree of vascular reactivity and the clinical severity of psoriasis. The more important factor seems to be disease duration, negatively correlating with changes in AChID and LTH, as well as with values of AChID and LTH at the second visit, after the LS diet.

To assess the impact of long-term, low-grade systemic inflammation on endothelial function in psoriasis, many researchers have examined the effects of inflammatory biomarkers, particularly CRP and hsCRP, with conflicting evidence in the literature [12]. In this study, no correlation was found between the inflammatory marker hsCRP and vascular reactivity.

This emphasizes the importance of early intervention targeting the restoration of endothelial function through primary and secondary prevention in PPs, which is still a reversible condition before irreversible atherosclerotic plaques and their calcification occur. As EDy occurs even in patients with milder disease where systemic treatment of psoriasis is not indicated, an LS diet could be a valuable addition to the holistic therapeutic approach in PPs.

For example, studies have shown that HS intake positively correlated with body weight and BMI, even after adjusting for calorie intake [53,54,55], and that waist circumference and body fat were higher in people with increased salt consumption [56]. Another study showed that subjects assigned to a 2-month LS diet showed reduced weight and BMI significantly more compared to a regular-salt diet group [57]. The results of the current study are consistent with those previously described, which could be attributed to a decrease in sodium intake and fluid retention.

In addition, lower BMI proved to be a predictor for greater recovery of endothelium-dependent vascular reactivity in PPs who followed an LS diet. One possible explanation is that the adipose tissue itself leads to a systemic pro-inflammatory state by promoting the secretion and release of pro-inflammatory mediators such as IL-6, IL-1β, TNF-α, leptin, and MCP-1, so that obesity per se is a risk factor for the development of EDy [58]. A 16-week LS diet resulted in decreased expression of MCP-1, IL-6, and TNF-α in mice, which was more than 50% lower in the adipose tissue of mice fed an LS diet than in mice fed an HS diet [59]. It could therefore be possible that this is a transitional finding and that a longer duration of the diet could improve endothelial function in people with a higher BMI. Furthermore, female sex was also a predictor for better AChID recovery. This is a very interesting finding since it is known that there are differences in the incidence, prevalence, and outcome of cardiovascular disease between men and women in the general population [60]. Furthermore, a recent study found that the salt sensitivity of blood pressure was 30% higher in women than in men, regardless of menopausal or hypertensive status [61]. These findings underscore the importance of considering sex differences when evaluating the impact of dietary salt on vascular health. It is important to point out that the exact mechanisms behind these sex differences are not yet fully elucidated and require further research. The interplay of genetics, hormones, vascular biology, and dietary salt intake likely plays a complex role in how an LS diet affects endothelial function differently in men and women [62].

Furthermore, in the present study, there was a negative correlation between baseline serum urate and the change in AChID before and after the LS diet. In general, there appears to be a correlation between elevated urate levels and psoriasis in Western Europe [63], which might be due to rapid cell turnover and long-term inflammation in psoriasis [64]. Also, high urate levels are considered an independent biomarker for more pronounced EDy in humans [65], and allopurinol therapy improves endothelial function [66]. Since in this study, PPs with higher baseline urate levels had smaller AChID changes after an LS diet, it is possible that salt is not involved in the mechanism of hyperuricemia-induced EDy in PPs.

In this study, the LS diet led to an increase in serum creatinine, although the values for both diets were within the reference intervals. At first glance, this result is surprising, as LS diets are thought to have a renoprotective effect [67]. This effect of LS diets has already been described in several studies. For example, a randomized study in people with chronic kidney disease showed that a low-salt diet led to a decrease in eGFR (estimated glomerular filtration rate) and consequently to an increase in serum creatinine and urate compared to a high-salt diet [68]. In addition, creatinine levels were lower in the salt-supplemented group than in the placebo group [69]. It appears that a high salt intake may lead to higher creatinine clearance, at least in the short term [68]. There was no statistically significant effect on serum urea, sodium, or potassium levels. Most of the available research in the literature does not describe such an effect of an LS diet.

This study has several limitations that should be acknowledged. First, it is a prospective cohort study with a single cohort, meaning there is no randomized control group for direct comparison, which may limit the strength of causal inferences. Second, the intervention period was relatively short, lasting only two weeks, which may not fully capture the long-term effects of salt restriction on endothelial function. Additionally, the sample size was relatively small, which may limit the generalizability of our findings. Also, we included patients with both psoriasis vulgaris and psoriatic arthritis, which could introduce bias, as psoriatic arthritis may have distinct vascular and inflammatory characteristics that differ from psoriasis alone. It is also worth mentioning that the above conclusions have been obtained within the limitations of the parameter space we have explored. Despite these limitations, our findings provide valuable insights into the potential benefits of salt reduction in this patient population. Future studies with larger, randomized controlled designs and longer intervention periods are needed to confirm these findings and further explore the potential sex-specific effects of salt restriction on microvascular function in psoriasis patients.

## 5. Conclusions

In conclusion, this study demonstrated a notable improvement in endothelium-dependent vascular reactivity among PPs following just two weeks on an LS diet. The findings highlight a negative correlation between the improvement in vascular reactivity and the duration of the disease, meaning that a longer disease duration was accompanied by a reduced reversibility of endothelial function. Importantly, significant sex differences were observed, with significant improvement in endothelial function being exclusive to women. Also, the improvement in endothelial function was greater in normotensive, normolipemic patients without central obesity.

To date, no official dietary recommendations for PPs have been published, while the importance of dietary lifestyle changes has been well recognized for many other diseases. This study highlights the importance of reducing salt intake in PPs and clarifies some of the positive effects. Overall, this shows that cutaneous microvascular reactivity in real-world PPs is complex and should be interpreted at multiple levels. Nevertheless, a direct test remains to be conducted, because machine learning algorithms fit data, not causality. As such, these methods may be suitable for extracting general and robust trends, but future studies with much larger samples will be necessary to build more realistic models.

## Figures and Tables

**Table 1:** Psoriasis severity and biochemical responses to a 2-week LS diet in psoriasis patients.

	RS	LS	*p* *
Clinical Psoriasis Characteristics			
Disease duration (years)	12.0 (7–26)		-
PASI	6.8 (5.1–11.2) ^†^	6.4 (5–9.5)	0.0011
Complete Blood Count			
Erythrocytes (×10^12^/L)	4.88 ± 0.39	4.89 ± 0.41	0.689
Thrombocytes (×10^9^/L)	256 (202–288)	235 (203–304)	0.958
Leukocytes (×10^9^/L)	7 ± 1.75	6.67 ± 1.75	0.321
Serum Biochemical Parameters			
Urea (mmol/L)	5.4 (4.5–6.1)	5.1 (4.6–5.8)	0.834
Creatinine (μmol/L)	71.4 ± 7.8	78.4 ± 12.7	0.0035
Sodium (mmol/L)	140 (139–140)	139 (138–140)	0.281
Potassium (mmol/L)	4.3 (4–4.7)	4.3 (4.1–4.5)	0.82
Calcium (mmol/L)	2.39 (2.35–2.42)	2.42 (2.4–2.46)	0.054
hsCRP (mg/L)	1.51 (0.8–2.7)	1.58 (0.61–2.89)	0.651
Fibrinogen activity (g/L)	3.5 ± 0.72	3.5 ± 0.78	-
Cholesterol (mmol/L)	4.93 ± 0.75	NA	-
Triglycerides (mmol/L)	1.41 ± 0.7	NA	-
HDL (mmol/L)	1.45 ± 0.28	NA	-
LDL (mmol/L)	3.24 ± 0.6	NA	-
HDL/LDL (%)	29 ± 5.88	NA	-
Biochemical parameters from 24 h urine samples			
24 h creatinine coefficient (μmol/kg/day)	154 (136–171)	134 (130–164)	0.313
24 h urine albumin (mg/dU)	5.6 (3.1–9.3)	5.7 (5.5–6.9)	0.835
24 h urine urea (mmol/dU)	326 (271–355)	284 (222.5–315.7)	0.455
24 h urine sodium (mmol/dU)	178.3 ± 58.6	83.1 ± 55.8	0.000064
24 h urine potassium (mmol/dU)	60.2 ± 17.1	58.6 ± 22.1	0.972
Calculated sodium intake (mg/day)	4101 ± 1348	1913 ± 1283	0.00064

RS—regular-salt diet; LS—low-salt diet; PASI—psoriasis area and severity index; hsCRP—high-sensitivity C-reactive protein; HDL—high-density lipoprotein; LDL—low-density lipoprotein; dU—diuresis; NA—not available. * Wilcoxon rank sum test. ^†^ median (interquartile range); otherwise, data are given as arithmetic mean ± standard deviation.

**Table 2:** Body shape indices and hemodynamic responses to a 2-week LS diet in psoriasis patients.

	RS	LS	*p* *
Anthropometric measures			
Body mass (kg)	82.9 ± 13.9	81.9 ± 13,3	0.00068
Height (cm)	174 ± 9.8	174 ± 10	1
Body mass index (kg/m^2^)	27.4 ± 4	27.2 ± 3.7	0.00081
Waist circumference (cm)	96.2 ± 14.3	95.5 ±13.3	0.0032
Hip circumference (cm)	105 ± 7.4	105 ± 6.5	0.145
Waist-to-hip ratio	0.91 ± 0.1	0.9 ± 0.09	0.148
Hemodynamics			
Systolic BP (mmHg)	126 ± 12.2	119 ± 12.9	0.021
Diastolic BP (mmHg)	82 ± 7.4	79 ± 8	0.079
Mean arterial pressure (mmHg)	97 ± 7.9	92 ± 8.3	0.0066
Heart rate (beats/min)	74 ± 14	77 ± 12	0.152
Laser Doppler flow measurement			
PORH 1 min. (n = 21)	100.6 ± 20.7 98.1 (84.9–114.6) ^†^	120 ± 33.4 115 (95.9–128.1) ^†^	0.056
AchID (Ach/B)	12.2 ± 5.2	14.8 ± 6.6	0.029
SNPID (SNP/B)	13.7 ± 4.9	12.8 ± 6.2	0.509
LTH (AUC)	9.9 ± 5.2	11.6 ± 6.8	0.297

RS—regular-salt diet; LS—low-salt diet. BP—blood pressure; SNPID (SNP/B)—dilation induced by sodium nitroprusside (flow after iontophoresis with sodium nitroprusside/basal flow); LTH—local thermal hyperemia; heat flow (increase)—increase in flow after local heating of the skin to 42 °C; AChID (Ach/B)—acetylcholine-induced dilation (flow after acetylcholine iontophoresis/basal flow); PORH 1 min AUC—post-occlusive reactive hyperemia after 1 min of occlusion (area under the curve). * Wilcoxon rank sum test. † median (interquartile range); otherwise, data are given as arithmetic mean ± standard deviation.

## Data Availability

The original contributions presented in this study are included in the article. The data that support the findings of this study are available from the corresponding authors upon reasonable request.

## Supplementary Materials

The following supporting information can be downloaded at https://www.mdpi.com/article/10.3390/nu17040693/s1, Figure S1. CONSORT diagram; Figure S2. Ridge plots showing an interaction between low-salt diet, hypertension, and central obesity; Figure S3. Non-parametric correlation analysis and acetylcholine-induced dilation measured after a 14-day low-salt diet; Figure S4. Shapley value decomposition and stacked force plots (acetylcholine-induced dilation after a 14-day low-salt diet) showing how each feature contributes to the model’s prediction for each participant (Lundberg–Erion–Lee prescription); Figure S5. Partial Spearman correlations for the net change (Δ) in acetylcholine-mediated vasodilation on a low-salt (LS) diet (significant residual associations (*p* < 0.05) after controlling for the baseline [z, AChID (regular-salt diet, RS)]); Figure S6. Correlation-based network and Spearman’s correlation coefficients (R); Figure S7. Predictive importance of each covariate and the non-parametric SHAP value decomposition for spectral analysis of the LDF-PORH signal (the frequency subinterval related to endothelial activity, the change between baseline values on a regular diet and values after a 14-day low-salt diet); Table S1: Demographic characteristics, life habits, comorbidities, and medication for PV subjects; Table S2. Hemodynamic responses to a 2-week LS diet in psoriasis patients (cutaneous laser Doppler flow measurements) split by a history of hypertension, central obesity, and hyperlipidemia; Table S3. Power spectrum of PORH-LDF signal response to a 2-week LS diet in psoriasis patients (n = 21).
