# Supplementary material for: Two-Week Low-Salt Diet Improves Acetylcholine-Induced Microvascular Dilation in Biologically Naïve Psoriasis Patients
Source: Nutrients. 2025 Feb 14;17(4):693. doi: 10.3390/nu17040693 (PMC11858809; doi:10.3390/nu17040693)
Supplement: Supplementary file 1 [file nutrients-17-00693-s001.zip › Supplementary figures and tables proofreading.pdf]

Supplementary Table S1. Demographic characteristics, life habits, comorbidities and medication, PV subjects.

Life habits	N (%)
N (Female/Male)	21 (12/9)
Age (years, mean ± SD)	46.7 ± 15.7
Smokers	5 (24%)
- years of smoking	25.4 ± 14
Former smokers	7 (33%)
- years of smoking	13.8 ± 8
- years since quitting	19.7 ± 13
Alcohol drinking regularly	3 (14%)
Comorbidities	
- Psoriatic arthritis	5 (25%)
- Arterial hypertension	7 (33%)
- Use of antihipertensive drugs	7 (33%)
- Myocardal infarction	1 (5%)
- Hyperlipidemia	11 (52%)
- Diabetes mellitus type II	1 (5%)
Previous psoriasis treatment	
Local corticosteroid treatment †	18 (86%)
Systemic drug use ‡	3 (14%)
- Methotrexate	1 (5%)
- Acitretine	1 (5%)
- Leflunomide	1 (5%)
Phototherapy ‡	3 (14%)
Biological therapy ‡	0 (0%)

† The subjects abstained from this treatment for ≥ 2 weeks.

‡ The subjects abstained from this treatment for ≥ 3 months.

Supplementary Table S2. Hemodynamic responses to a 2-week LS diet in psoriasis patients (cutaneous laser Doppler flow measurements) split by a history of hypertension, central obesity, and hiperlipidemia.

PORH 1 min. (AUC)	RS	LS	p*	HL (95 % CI)
Normotensive (n=14)	92.6 (83-109.8)	114.8 (96.1-127)	0.02	17.9 (3.2, 57.7)
Hypertensive (n=7)	118.6 (96.1-127)	118.9 (92.8-129.8)	0.272	-0.2 (-35.3, 45.4)
CO=NO (n=9)	92.8 (74.7-110.5)	124 (109.6-150.6)	0.032	37.4 (3.7, 81.3)
CO=YES (n=12)	106.1 (88.7-117.9)	112.7 (88.3-126.5)	0.61	2.2 (-17.1, 21.1)
HL=NO (n=10)	90 (79.2-110)	119.5 (104-137.3)	0.025	28.9 (5.8, 61.6)
HL=YES (n=11)	110.8 (92.8-127.3)	113.5 (90.7-129)	0.76	1.9 (-20.7, 33.5)
PORH-ET (%)	RS	LS	p*	HL (95 % CI)
Normotensive (n=14)	61.2 (49.5-71.5)	73.3 (67.6-79.3)	0.0057	10.7 (4, 22.1)
Hypertensive (n=6)	72.6 (56.4-75.5)	61.7 (55.4-75.6)	0.295	-4 (-19, 7)
CO=NO (n=9)	64.2 (48.1-73.4)	77.7 (67.2-81.6)	0.017	10.9 (3.3, 24.3)
CO=YES (n=11)	61.7 (57.1-75.1)	68.6 (56.1-75)	0.965	0.3 (-5.8, 17.5)
HL=NO (n=10)	61.2 (51.8-69.7)	73.3 (66.1-78.5)	0.025	9.3 (1.9, 17.8)
HL=YES (n=10)	72.7 (53.5-77.6)	66.7 (60-78.6)	0.61	2.2 (-5.8, 19.6)
AChID	RS	LS	p*	HL (95 % CI)
CO=NO (n=9)	11.4 (8.5-18.1)	20 (11.9-23.6)	0.033	4.3 (0.5, 10.1)
CO=YES (n=12)	12.6 (7-13.9)	11.5 (7.9-15.3)	0.456	1.1 (-1.6, 3.4)
HL=NO (n=10)	12.6 (9.2-18)	19.8 (12.2-22.3)	0.014	4.2 (1.1, 9.6)
HL=YES (n=11)	11.4 (6.9-15.1)	11.1 (7.7-15.1)	0.824	0.2 (-1.8, 3.2)

RS – regular salt diet; LS – low salt diet; CO – central obesity; HL – hyperlipidemia; HL – Hodges-Lehmann estimate of treatment effect; CI – confidence interval. PORH 1 min AUC – post-occlusive reactive hyperemia after 1 minute of occlusion (area under the curve); AChID (Ach/B) – acetylcholine-induced dilation (flow after acetylcholine iontophoresis / basal flow); PORH-ET – spectral analysis of the LDF-PORH signal, the frequency subinterval related to endothelial activity. Data are presented as median (interquartile range).

* Wilcoxon rank sum test.

Supplementary Table S3. Power spectrum of PORH-LDF signal response to a 2-week LS diet in psoriasis patients (n=21).

% of total power within frequency band	RS	LS	p*
Cardiac (0.6–2.0 Hz)	11 (5.2 - 19.1)†	8.9 (3.1 - 18.6)	0.151
Respiratory (0.15–0.6 Hz)	6.97 ± 5.48	5.24 ± 4.23	0.36
Myogenic (0.05–0.15 Hz)	4.72 ± 4.47	4.22 ± 3.08	0.94
Sympathetic (0.02–0.05 Hz)	11.00 (7.8 - 13.2)	9.60 (8.35 - 12.60)	0.332
Endothelial (0.008–0.02 Hz)	63 (54.1 - 75.1)	72.2 (61.5 - 78.3)	0.038

PORH- post-occlusive reactive hyperemia; LDF – laser Doppler flowmetry; RS – regular salt diet; LS – low salt diet.

* Wilcoxon rank sum test.

† median (interquartile range), otherwise data are given as arithmetic mean ± standard deviation.

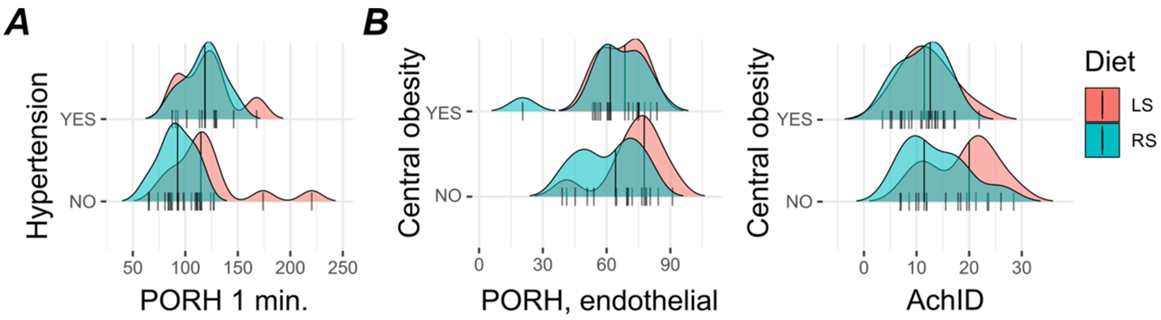

Supplementary Figure S1. Ridge plots, showing an interaction between low-salt diet, hypertension (panel A) and central obesity (panel B).

The shaded regions denote data density, the median is indicated by the vertical dashed black lines. The number of participants in each class is shown by marginal distribution along the x-axis.PORH 1 min – post-occlusive reactive hyperemia after 1 minute of occlusion (area under the curve); PORH-endothelial – spectral analysis of the LDF-PORH signal, the frequency subinterval related to endothelial activity; AChID (Ach/B) – acetylcholine-induced dilation (flow after acetylcholine iontophoresis / basal flow).

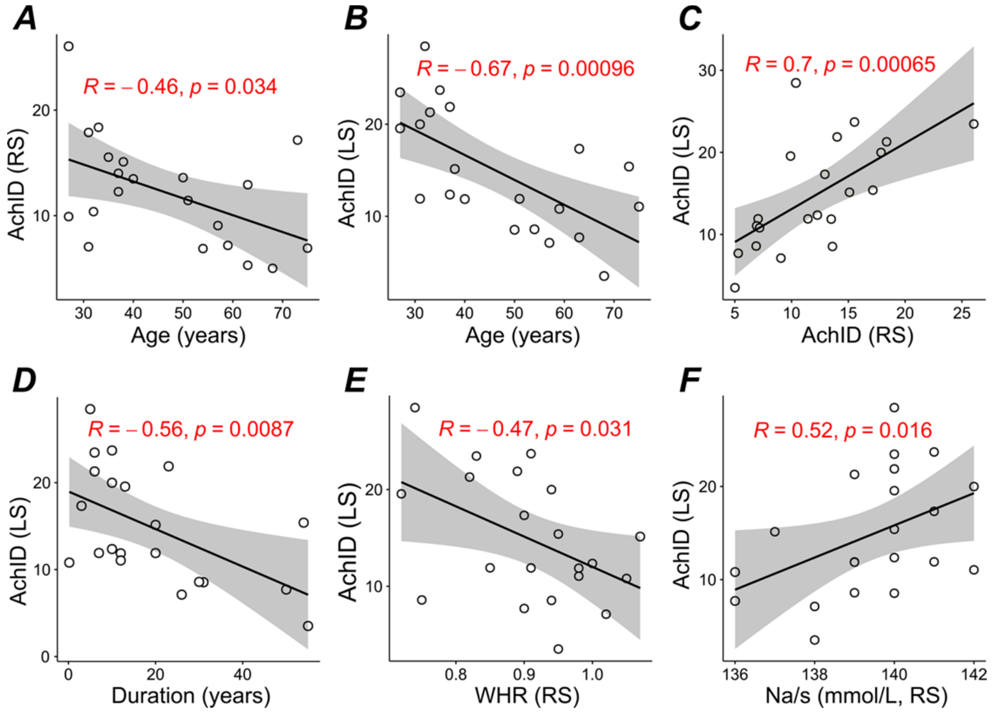

Supplementary Figure S2. Nonparametric correlation analysis, acetylcholine-induced dilation measured after a 14-day low-salt diet.

R denotes Spearman's correlation coefficient. The shaded area around the black regression line (least squares solution) represents the 95% confidence interval. Each point corresponds to one subject. AChID – Acetylcholine-induced dilation (flow after acetylcholine iontophoresis / basal flow); RS – regular salt diet, LS – low-salt diet; WHR – waist-to-hip ratio.

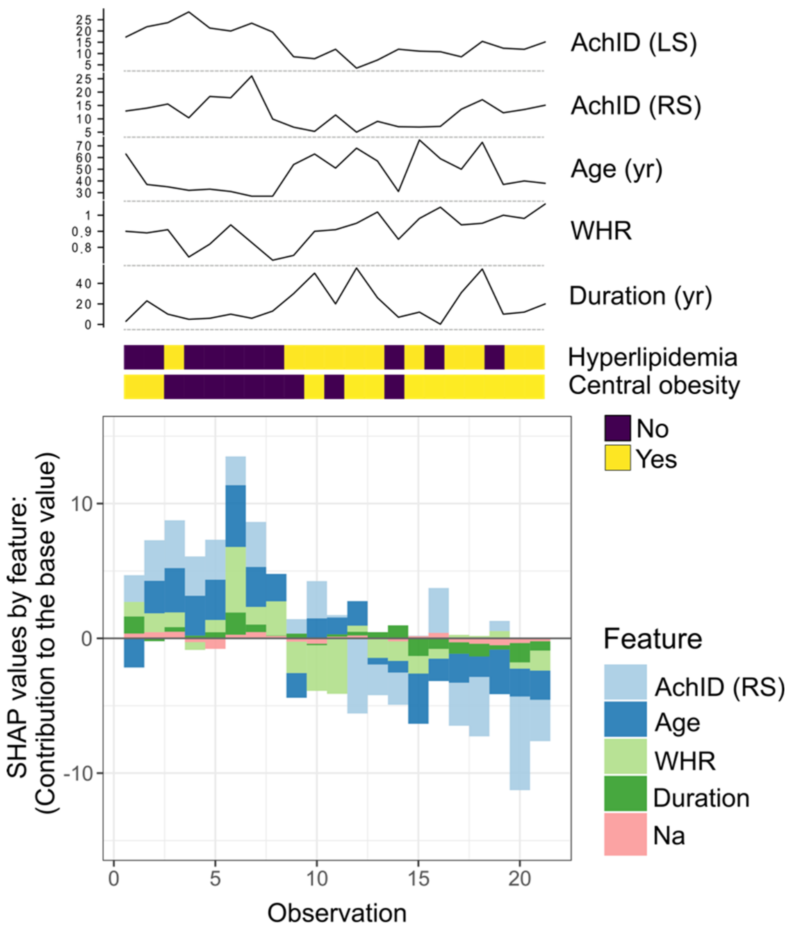

Supplementary Figure S3. Shapley value decomposition, stacked force plots (acetylcholine-induced dilation after a 14-day low-salt diet) showing how each feature contributes to the model's prediction for each participant (Lundberg-Erion-Lee prescription, Ref. [35]). Observations (participants) are ordered by the similarity of their SHAP values (hierarchical clustering, Ward.D method), identifying trends and patterns in feature contributions across a dataset. The SHAP values indicate the direction of the relationship (positive or negative) between the predictive feature and the target variable (AChID-LS), the size of the impact is represented by the size of the bar. Heatmap-like graphics and line annotations depict the corresponding feature values across the entire dataset.

AChID – Acetylcholine-induced dilation (flow after acetylcholine iontophoresis / basal flow); RS –regular salt diet; LS – low-salt diet; WHR – waist-to-hip ratio; Na – serum sodium levels.

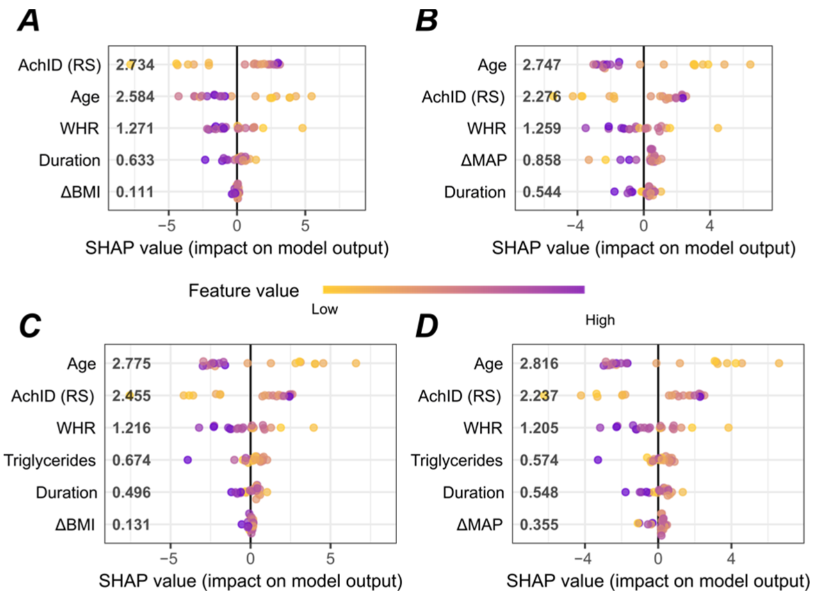

Supplementary Figure S4. Sensitivity analysis, acetylcholine-induced dilation (AChID) measured after a 14-day low-salt (LS) diet.

The predictive value (relative contribution) of each covariate (Shapley value decomposition) is shown. The impact of the top four predictors (Figure 4A) was reassessed by adjusting the model for changes (Δ) in body mass index (BMI, panel A) and mean arterial blood pressure (MAP, panel B) between the two time points. In the next step, the two models were further extended by the addition of triglycerides to the predictor list (panels C–D). Features were ranked vertically by their mean absolute SHAP values over all observations (numbers on the right of the variable names, the larger the absolute SHAP value the greater the importance of the predictor for the model's output). Clusters of dots around the SHAP value of zero indicate a small impact on model output; each dot corresponds to one participant. The color is scaled to the feature value from low to high. RS – regular salt diet; WHR – waist-to-hip ratio; ΔBMI = BMI (LS) - BMI (RS); ΔMAP = MAP (LS) - MAP (RS).

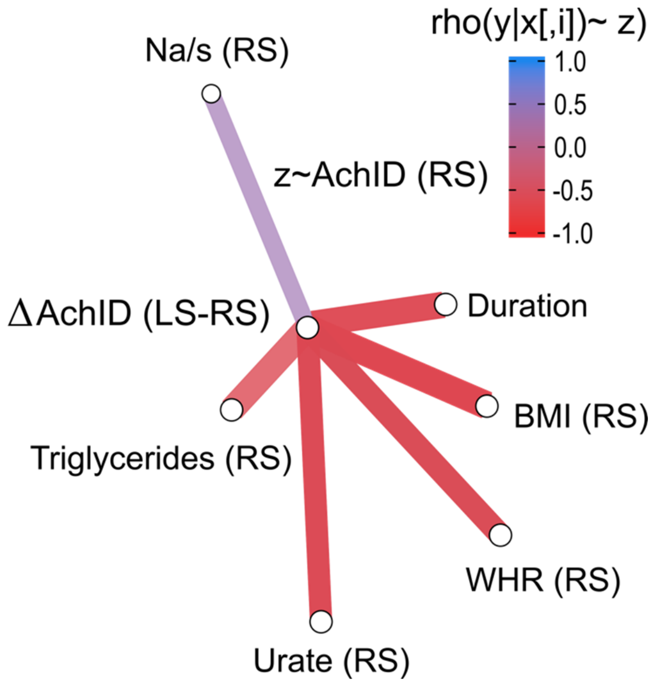

Supplementary Figure S5. Partial Spearman correlations for the net change (Δ) in acetylcholine-mediated vasodilation on a low-salt (LS) diet (significant residual associations ($p<0.05$) after controlling for the baseline [z, AChID (regular salt diet, RS)]). The colored vertical band and lines encode the partial correlation coefficient (rho).

AChID – Acetylcholine-induced dilation (flow after acetylcholine iontophoresis / basal flow); WHR – waist-to-hip ratio; BMI – body mass index. Na/s – serum sodium levels.

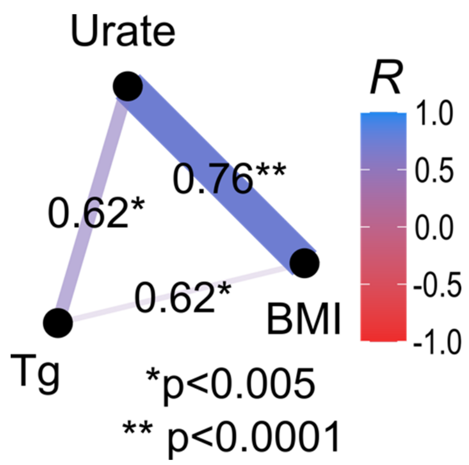

Supplementary Figure S6. Correlation-based network, Spearman's correlation coefficients (*R*). Blue and red colors at the edge denote positive and negative modes, respectively. The width and transparency of the edges are proportional to the strength of the correlation. Tg – serum triglycerides; BMI – body mass index.

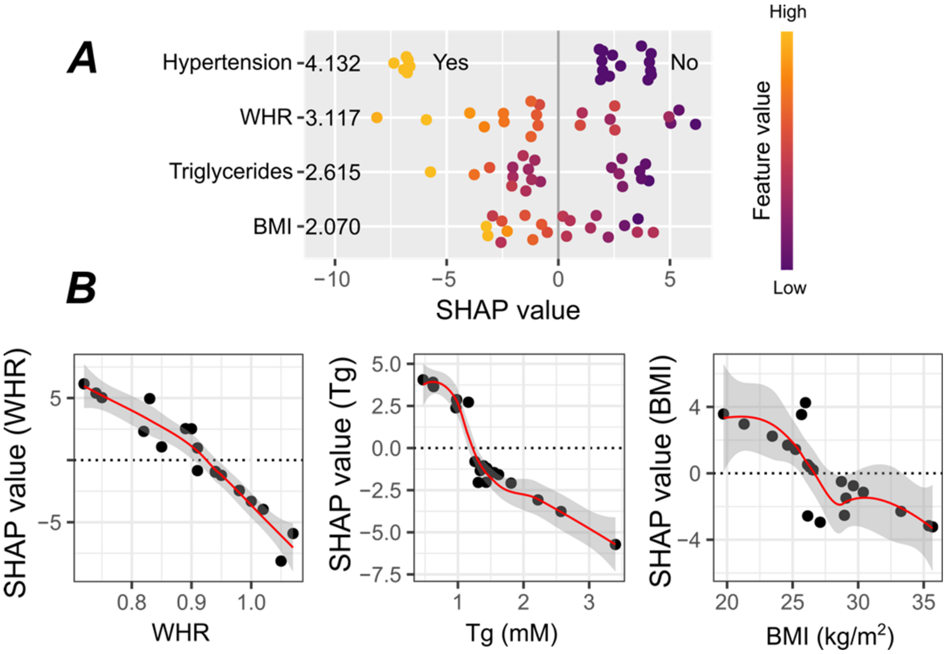

Supplementary Figure S7. Predictive importance of each covariate, non-parametric SHAP value decomposition for spectral analysis of the LDF-PORH signal (the frequency subinterval related to endothelial activity, the change between baseline values on a regular diet and values after a 14-day low-salt diet).

A) Predictive importance of each covariate. Summary of SHAP values for every predictor considered in this analysis. Features were ranked by their mean absolute SHAP values over all observations (bold on the right of the variable names, the larger the absolute SHAP value the greater the importance of the predictor for model's output). Each dot corresponds to one participant. The color is scaled to the feature value from low to high.

B) Partial dependence plots, showing how the impact of feature changes as the feature value changes. The feature of interest is represented along the horizontal axis, while the corresponding SHAP values are plotted on the y-axis. For average first-order effect, a non-parametric locally weighted running line smoother (LOESS) was fitted to visualize trends in data (shade represents standard error). WHR – waist-to-hip ratio; Tg – serum triglycerides; BMI – body mass index; mM – mmol/L.
