# Supplementary figures and images for: Two-Week Low-Salt Diet Improves Acetylcholine-Induced Microvascular Dilation in Biologically Naïve Psoriasis Patients
Source: Nutrients. 2025 Feb 14;17(4):693. doi: 10.3390/nu17040693 (PMC11858809; doi:10.3390/nu17040693)

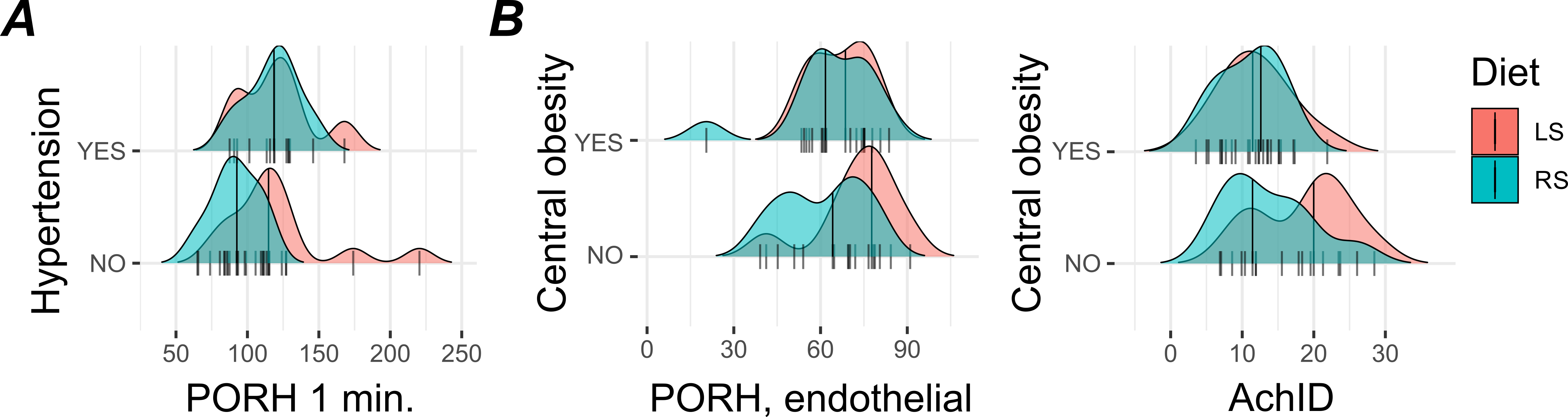

Supplement: Supplementary file 1 [file nutrients-17-00693-s001.zip › SF1R4.png]

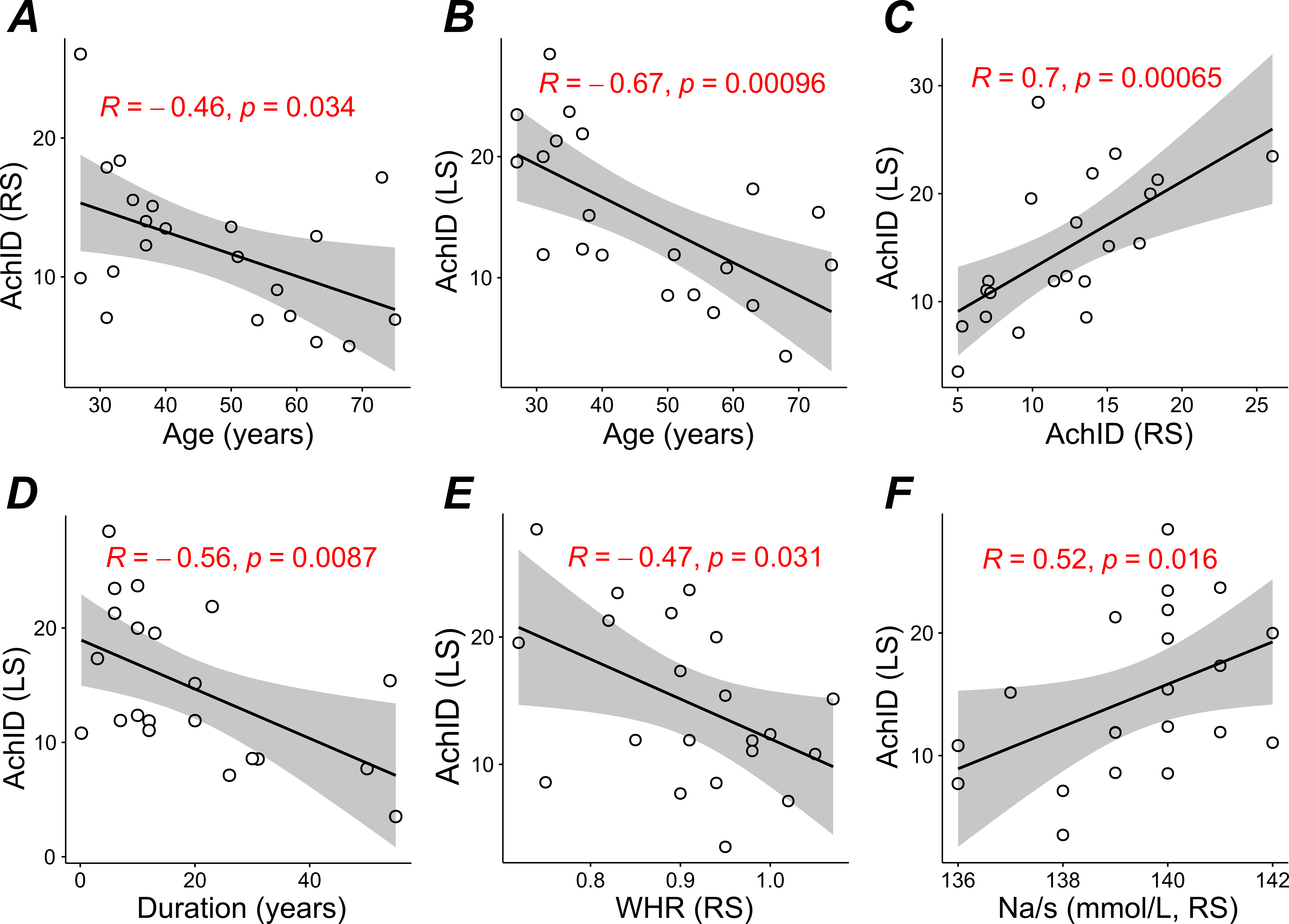

Supplement: Supplementary file 1 [file nutrients-17-00693-s001.zip › SF2R4.png]

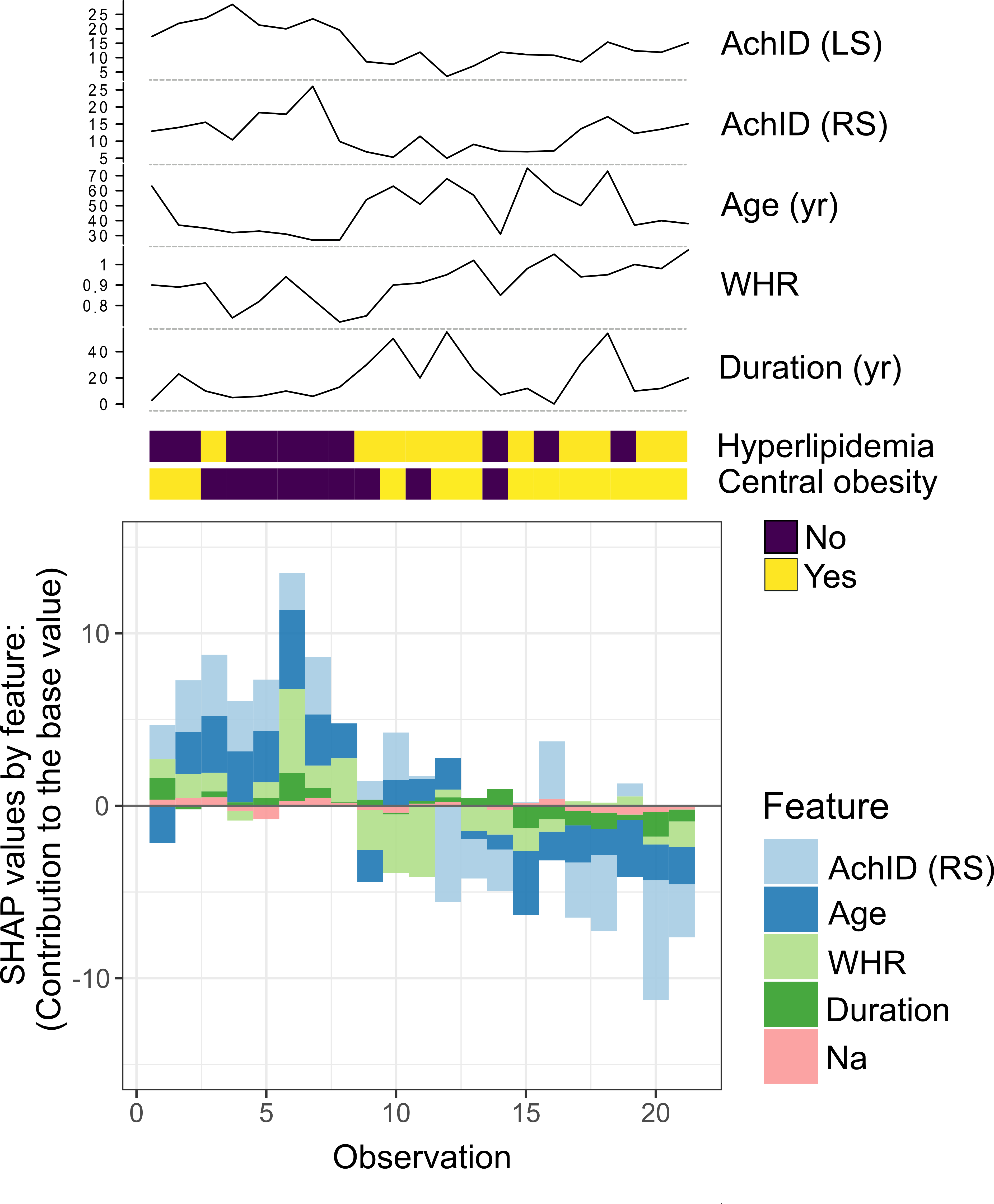

Supplement: Supplementary file 1 [file nutrients-17-00693-s001.zip › SF3R4.png]

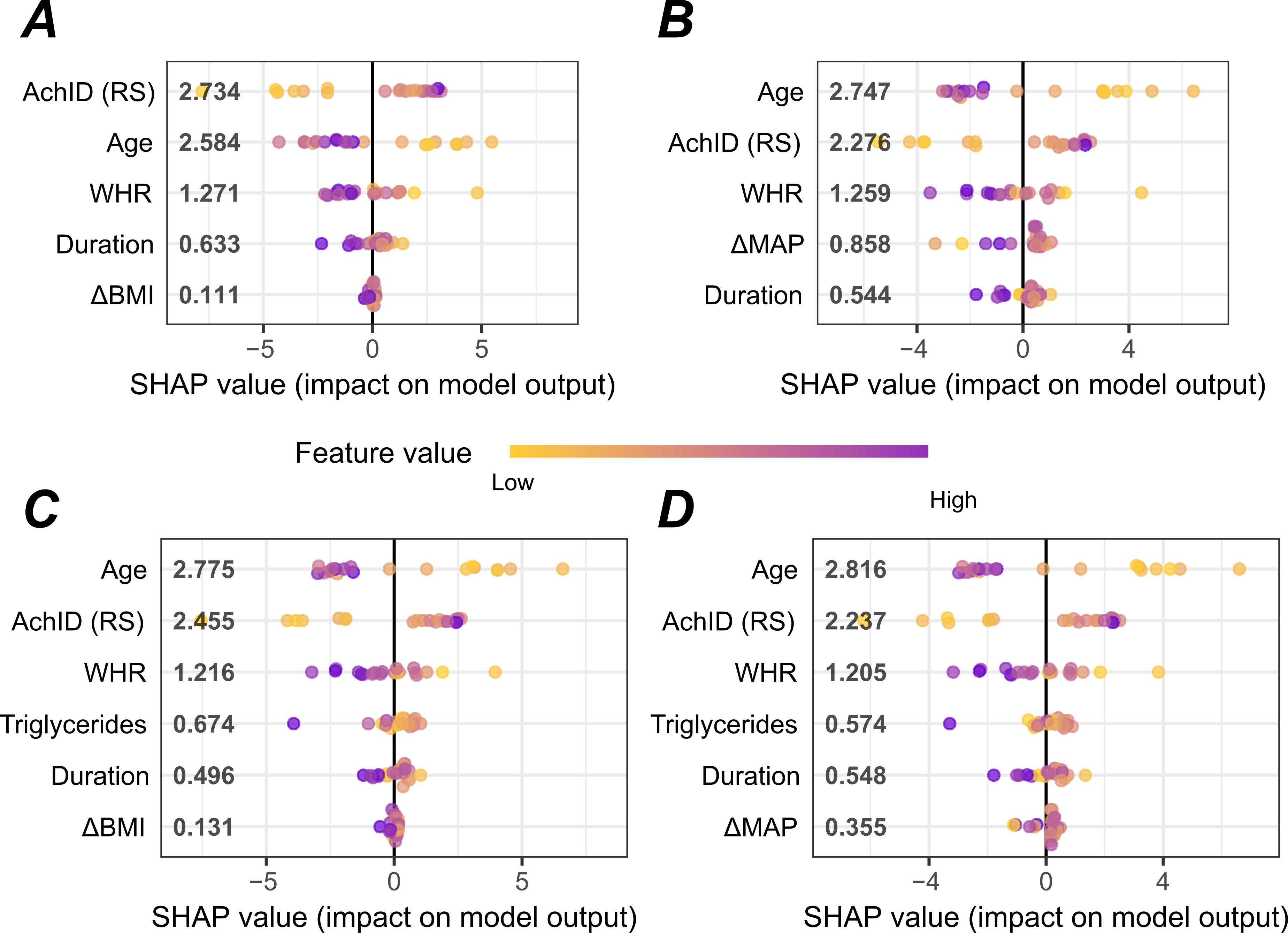

Supplement: Supplementary file 1 [file nutrients-17-00693-s001.zip › SF4H.png]

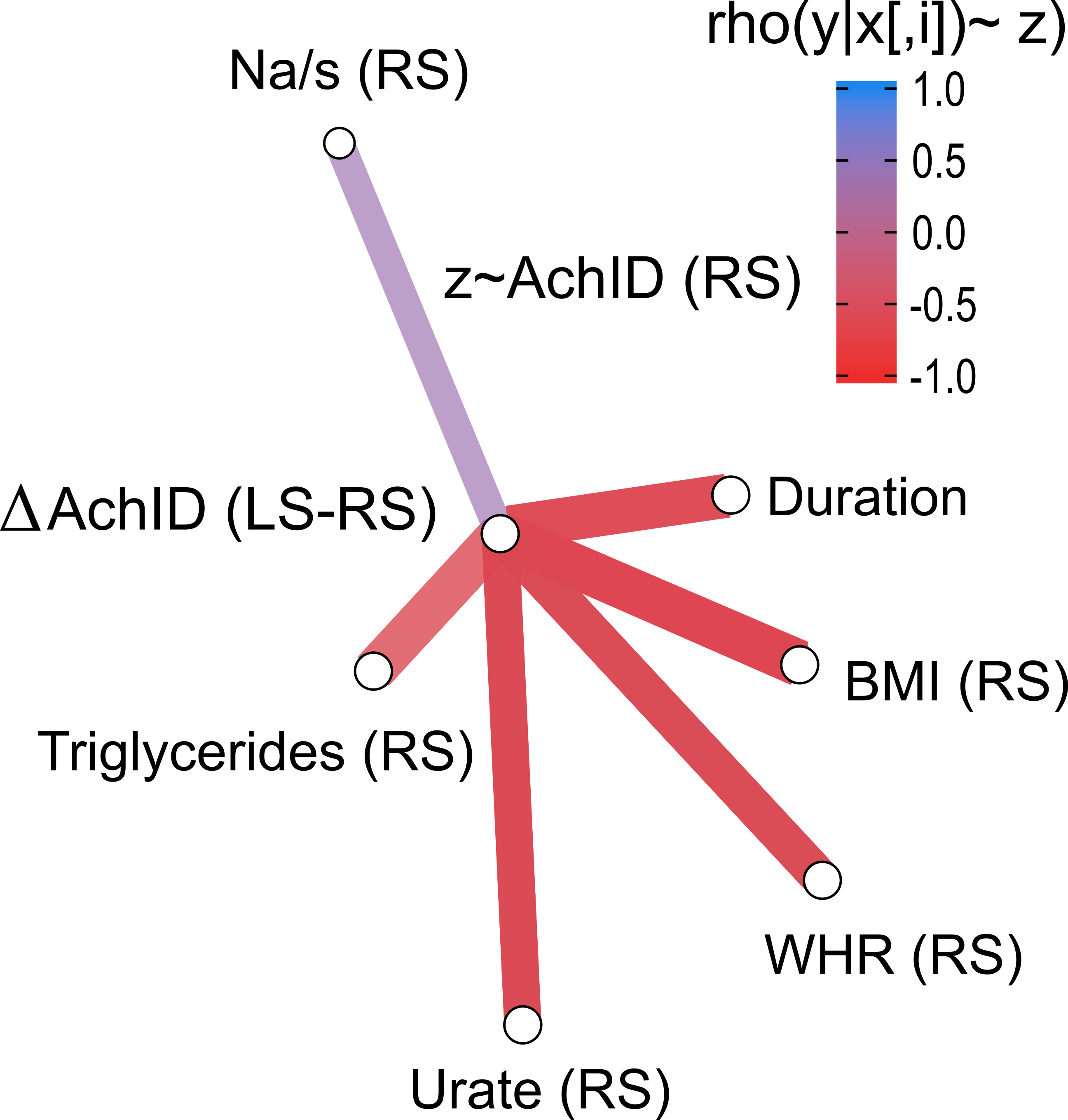

Supplement: Supplementary file 1 [file nutrients-17-00693-s001.zip › SF5R4.png]

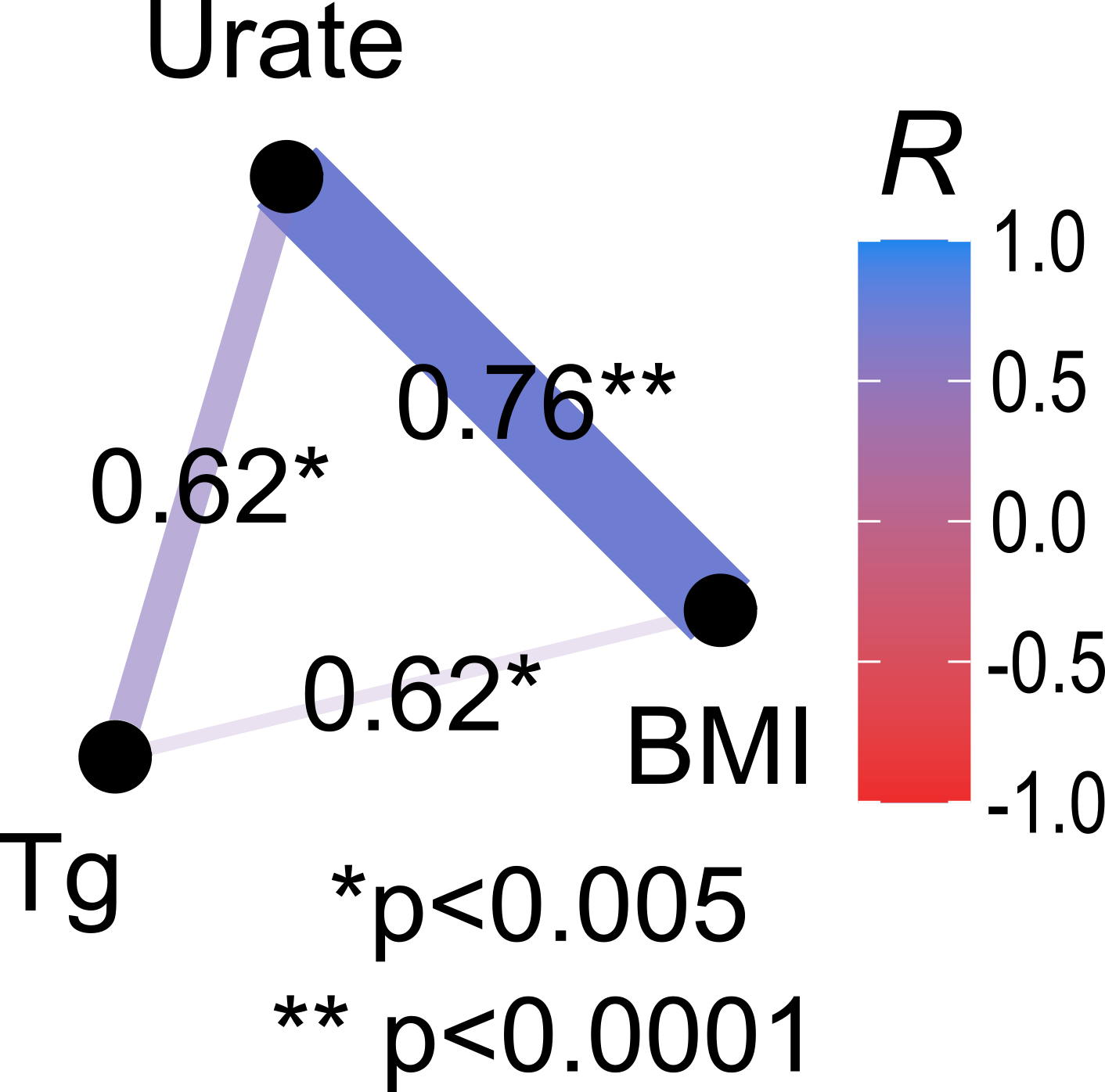

Supplement: Supplementary file 1 [file nutrients-17-00693-s001.zip › SF6R4.png]

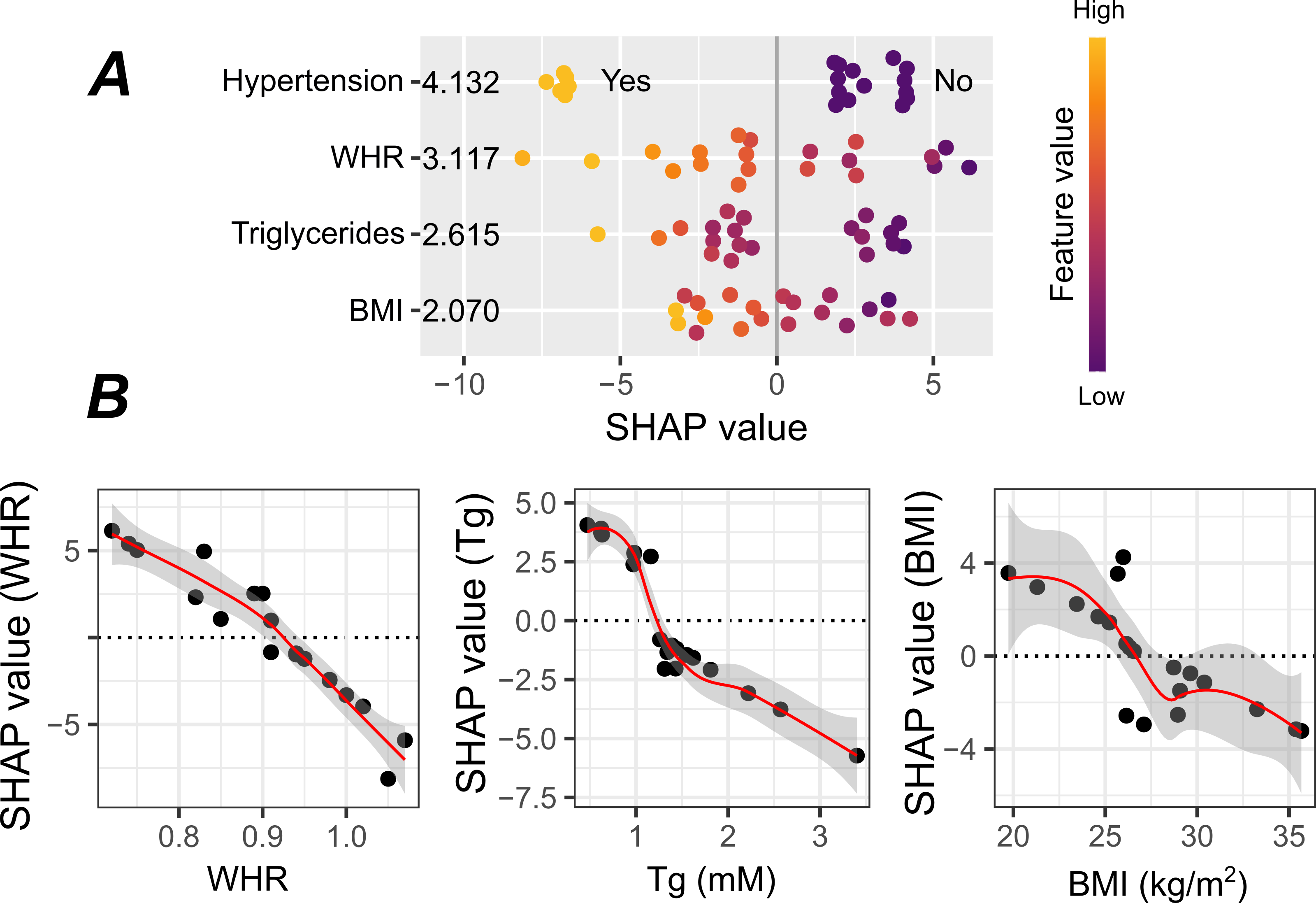

Supplement: Supplementary file 1 [file nutrients-17-00693-s001.zip › SF7H.png]
